# Microbiota Inhibit Epithelial Pathogen Adherence by Epigenetically Regulating C-Type Lectin Expression

**DOI:** 10.3389/fimmu.2019.00928

**Published:** 2019-05-07

**Authors:** Vivienne Woo, Emily M. Eshleman, Taylor Rice, Jordan Whitt, Bruce A. Vallance, Theresa Alenghat

**Affiliations:** ^1^Division of Immunobiology and Center for Inflammation and Tolerance, Cincinnati Children's Hospital Medical Center, Department of Pediatrics, University of Cincinnati College of Medicine, Cincinnati, OH, United States; ^2^Division of Gastroenterology, Department of Pediatrics, BC Children's Hospital Research Institute, University of British Columbia, Vancouver, BC, Canada

**Keywords:** microbiota, HDAC, CLEC, intestine epithelial cells, citrobacter, epigenetic

## Abstract

Numerous bacterial pathogens infect the mammalian host by initially associating with epithelial cells that line the intestinal lumen. Recent work has revealed that commensal bacteria that reside in the intestine promote defense against pathogenic infection, however whether the microbiota direct host pathways that alter pathogen adherence is not well-understood. Here, by comparing germ-free mice, we identify that the microbiota decrease bacterial pathogen adherence and dampen epithelial expression of the cell surface glycoprotein C-type lectin 2e (Clec2e). Functional studies revealed that overexpression of this lectin promotes adherence of intestinal bacterial pathogens to mammalian cells. Interestingly, microbiota-sensitive downregulation of Clec2e corresponds with decreased histone acetylation of the Clec2e gene in intestinal epithelial cells. Histone deacetylation and transcriptional regulation of Clec2e depends on expression and recruitment of the histone deacetylase HDAC3. Thus, commensal bacteria epigenetically instruct epithelial cells to decrease expression of a C-type lectin that promotes pathogen adherence, revealing a novel mechanism for how the microbiota promote innate defense against infection.

## Introduction

Infections of the gastrointestinal tract are a major cause of morbidity and mortality worldwide. Specifically, enteric infections caused by bacterial pathogens account for well over 200 million individual cases of enteritis resulting in an estimated 5 million deaths annually (1, 2). In addition to local intestinal infections, the gastrointestinal tract is the initial site of adhesion and entry for several pathogens that disseminate to cause systemic disease (3). Thus, adherence and invasion are critical steps in the pathogenesis of both enteric and systemic bacterial infections. In order to establish disease, pathogens can interact with host cells by expressing adhesin molecules which recognize various components such as extracellular matrix proteins, integral membrane adhesion receptors, and cell membrane associated glycoproteins (4). These interactions between bacterial pathogens and host cells are not only critical for initiating infection, but also direct tissue tropism, species specificity, and host susceptibility to infection (4–7). Therefore, understanding how pathogenic adherence is mediated is critical for directing effective approaches that prevent and treat enteric infections.

In addition to pathogenic bacteria, the mammalian gastrointestinal tract harbors trillions of innocuous commensal bacteria. These commensal microbes, collectively termed the microbiota, are required for healthy intestinal development and immune cell activation (8). Importantly, the presence of the microbiota has also been consistently shown to be essential for host defense against pathogenic infections (8, 9). While several mechanisms have been proposed to account for microbiota-dependent protection against infection, many pathways indicate that commensal bacteria can potentiate host-cell intrinsic defenses (10–12). Intestinal epithelial cells (IECs) reside at the direct interface between the microbiota and underlying host immune cells and are in constant contact with both beneficial as well as invading bacteria. Thus, IECs are a key cell type to which enteric pathogens often directly associate with in order to infect and invade the host. In addition to mediating binding and sensing of microbial components, these critically located cells can actively respond to pathogenic challenges by secreting antimicrobial peptides, mucins, and cytokines that prime and regulate innate and adaptive immune cell compartments (13–16). However, it is not clear whether the microbiota restrict enteric infection by regulating pathogen binding to the intestinal epithelium.

In mammalian cells, DNA is packaged around histone proteins that are condensed into a higher order structure called chromatin. In general, chromatin structure itself restricts access of transcriptional machinery to the genome thereby repressing gene expression. However, covalent modifications of the amino-terminal tails of histones, specifically, acetylation, methylation, phosphorylation, SUMOylation, and ubiquitination are associated with conformational changes in the chromatin landscape. For example, histone acetylation is known to generate an open chromatin structure that contributes to active transcription (17–19). These modifications are mediated by epigenetic modifying enzymes such as histone acetyltransferases and histone deacetylases (HDACs). The balance and pattern of these modifications on specific histone tails regulate chromatin reorganization and direct transcriptional machinery. Thus, epigenetic modifications enable environmental signals to trigger transcriptional changes without altering underlying DNA sequence (20–22).

In this study, we aimed to test whether the microbiota affect IEC-intrinsic pathways that alter the ability of pathogens to adhere to the IECs. *Citrobacter rodentium*, a murine enteric pathogen with a similar pathogenesis to enteropathogenic *E. coli* in humans, infects the host by initially adhering to IECs. By employing germ-free (GF) mice, we identified that the microbiota reduce pathogen colonization with *C. rodentium* during infection and instruct decreased IEC interactions with the pathogen. Global gene expression analyses revealed that the microbiota highly suppressed IEC expression of the cell-surface C-type lectin 2e (Clec2e). Interestingly, functional studies showed that overexpression of Clec2e enhanced pathogen bacterial binding to the mammalian cell membrane. Furthermore, microbiota-dependent transcriptional suppression of Clec2e in IECs correlated with decreased histone acetylation and recruitment of the histone deacetylase, HDAC3. Collectively, these data demonstrate a novel mechanism by which commensal bacteria in the intestine epigenetically regulate expression of a pathogen-binding glycoprotein to promote host defense against infection.

## Materials and Methods

### Mice and *in vivo* Infections

Conventionally-housed C57Bl/6J mice were purchased from Jackson Laboratories and maintained in our specific-pathogen free colony at CCHMC. Germ-free (GF) mice were maintained in plastic isolators in the CCHMC Gnotobiotic Mouse Facility, fed autoclaved feed and water, and monitored to ensure absence of microbes. HDAC3^FF^ mice (23) were crossed to C57Bl/6J mice expressing Cre recombinase under control of the IEC-specific villin promoter (24) to generate HDAC3^Δ*IEC*^ mice (25). Mice were housed up to 4 per cage in a ventilated cage system in a 12 h light/dark cycle, with free access to water and food. For *C. rodentium* infection, age- and gender- matched mice were orally inoculated with 10^9^ colony forming units (CFUs) of *C. rodentium* (26, 27). To enumerate intestinal bacterial burdens, stool was collected in PBS and homogenized in a TissueLyser II at 30 Hz for 3 min. Homogenates were serially diluted and plated on MacConkey agar. CFUs were counted and normalized to stool weight after 18 h. All experiments were performed according to the animal guidelines upon approval of the Institutional Animal Care and Use Committee at CCHMC.

### IEC Harvest, RNA Analyses, Western Blotting

IECs were harvested from mouse intestine as described previously (25, 27, 28). IECs from the small intestine were harvested from the most distal 12 cm section. RNA was isolated from cells using the RNeasy Kit (Qiagen) then subjected to reverse transcription with Verso reverse transcriptase (Thermo Fisher). Directional polyA RNA-seq for IECs from the small intestine was performed by the Sequencing Core at the University of Cincinnati (28). Sequence reads were aligned by using Illumina sequence analysis pipeline by the Laboratory for Statistical Genomics and Systems Biology at the University of Cincinnati. Real-time PCR was performed using SYBR (Applied Biosystems) and analyzed with a threshold in the linear range of amplification using primer sequences as follows: Clec2eF: 5′-AGCAAGGTTCACAGCTCTCC-3′; Clec2eR: 5′-GCTGCTATGGAGTGATCATGG-3′; RegIIIγF: 5′-TTCCTGTCCTCCATGATCAAA-3′; RegIIIγR: 5′-CATCCACCTCTGTTGGGTTC-3′; HPRTF: 5′-GATTAGCGATGAACCAGGT-3′; HPRTR: 5′-CCTCCCATCTCCTTCATGACA-3′. Expression analysis in IECs from large intestine of HDAC3^FF^ and HDAC3^Δ*IEC*^ mice by microarray was described previously (25). For western blot analyses, total cell lysates were probed with anti-histone H3 (Santa Cruz) and anti-DDK (FLAG) (Origene) and imaged using an Odyssey Fc imager (LICOR). Global expression data has been deposited in NCBI's Gene Expression Omnibus (GEO) and is accessible through accession number GSE128362.

### ChIP-Sequencing

ChIP was performed as described previously with few modifications (29). Briefly, cells were fixed in 1% PFA for 10 min and quenched with glycine. Total cell extracts were sonicated using a Covaris S220 Focused-ultrasonicator and nuclear extracts were immunoprecipitated with rabbit anti-H3K9Ac (Millipore, 06-942) or rabbit anti-HDAC3 (Abcam, ab7030) using a SX-8G IP-STAR robot. Sequencing was performed using Illumina HiSeq 2500, mapped to mus musculus genome mm10 with Bowtie and peaks were identified with MACS (30, 31) and visualized in Biowardrobe (32). ChIP-qPCR was performed using SYBR (Applied Biosystems) and analyzed as fold difference normalized to an unaffected control gene. Reactions were run on a real-time PCR system (QuantStudio3; Applied Biosystems) with custom made primer pairs: Clec2e-ChIPF: 5′-ACACAAGATGCAGCGGAGAT-3′; Clec2e-ChIPR: 5′-GTGAAGGGGTTTTCACTAGGGG-3′; Insl-ChIPF: 5′-CAGAGACCATCAGCAAGCAG-3′; Insl-ChIPR: 5′-TTCTCCCTAAAGTCGCTGGA-3′; Albumin-ChIPF: 5′-AGAGCGATCTTTCTGCACACA-3′; Albumin-ChIPR: 5′-AGGAGAAAGGTTACCCACTTCATTT-3′. ChIP-seq data is accessible through GEO series accession numbers GSE50453 and GSE128369.

### Cell Culture and Immunofluorescence

HEK293T cells were cultured in DMEM containing 10% FBS, 100 U/ml penicillin, and 100 mg/ml streptomycin at 37°C and 5% CO_2_. Cells were transiently transfected with pCMV6-Clec2e-myc-DDK (FLAG) vector (Origene, MR202134) using Lipofectamine 3000 (Thermo Fisher). Transfected cells were seeded onto Retronectin (Takara Bio) coated chamber slides (Ibidi) and infected with 10^6^ CFUs of GFP-expressing *C. rodentium* for 6 h in antibiotic-free media (26, 27). Cells were washed in PBS 3 times and fixed in 4% paraformaldehyde for 20 min. Fixed cells were blocked with 2% BSA for 1 h at room temperature and stained in 0.5% BSA with 488-anti-GFP (Thermo Fisher, 1:300), Phalloidin (Invitrogen, 1:200) and DAPI (Invitrogen, 1:1,000) for 1 h at room temperature. Stained cells were visualized using Nikon A1R LUN-V inverted confocal microscope.

### Intestinal Organoids

Murine organoids were generated from colonic crypts isolated from germ-free and conventionally-housed mice as previously described (33). Briefly, dissected colons were opened longitudinally, scrapped to remove intestinal contents and outer cells, washed repeatedly in ice-cold PBS, and cut into 1-cm pieces. Colonic pieces were incubated in chelation buffer (2 mM EDTA in PBS) for 30 min at 4°C with rotation. The tissue was transferred into a new tube containing Shaking Buffer (PBS, 43.3 mM sucrose, 54.9 mM sorbitol) and gently shaken by hand for 2–4 min. Colonic crypts were resuspended and plated in Matrigel (Corning) with organoid culture media (60% Advanced DMEM/F12 media supplemented with 10 mM HEPES, 2 mM L-glutamate, 40% L-WRN conditioned media, 1x N2 supplement, 1x B27 supplement, 50 ng/mL murine EGF, and 10 μM Y-27632 ROCK inhibitor) overlaid. Culture media was changed every 3–4 days. Organoid cultures were infected with GFP-*C. rodentium* at a concentration of 10^6^ CFUs for 24 h. After incubation, organoids were washed 3 times in ice-cold PBS, dislodged from plate and Matrigel, and fixed in 1% PFA for 1 h at 4°C. GF organoids were stimulated with 10 ng/mL of *E. coli* LPS for 24 h.

### Flow Cytometry

Cells were stained for flow cytometry using the following fluorescence-conjugated antibodies diluted in FACS Buffer (2% FBS, 0.01% Sodium Azide, PBS): PE anti-CD326 (EpCAM) (Clone: G8.8, eBioscience), BUV395 anti-CD45.2 (Clone: 104, BD Biosciences), 488 anti-GFP (Clone; FM264G, BioLegend). Dead cells were excluded with the Fixable Violet Dead Cell Stain Kit (Invitrogen). Samples were acquired on the BD LSRFortessa and analyzed with FlowJo Software (Treestar). The geometric mean fluorescence intensity (MFI) for GFP-*C. rodentium* expression was assessed and the background MFI determined in uninfected controls was subtracted from infected samples.

### Bacterial Adhesion Assay

Adhesion of pathogenic bacteria to mammalian cells was determined as previously described (34). Briefly, HEK293T cells were seeded at 70–90% confluency and incubated with GFP-*C. rodentium* or wild-type *Salmonella enterica* serovar Typhimurium at a multiplicity of infection (MOI) of 5:1 (bacteria:cells) for 6 h in antibiotic-free media. Cells were washed 3 times with PBS and adherent bacteria were detached using a 1% triton-X 100 lysis buffer and plated onto MacConkey agar in 10-fold serial dilutions. Colony forming units (CFUs) were counted after 16 h.

### Statistical Analyses

Results are expressed as mean ± SEM. Statistical significance was determined with the Student's *t*-test, with all data meeting the assumptions of the statistical test used. Results were considered significant at ^*^p < 0.05; ^**^p < 0.01; ^***^p < 0.001. Statistical significance was calculated using Prism version 7.0 (GraphPad Software).

## Results

### Microbiota Decrease Pathogen Adherence to Intestinal Epithelial Cells

**Figure 1:**
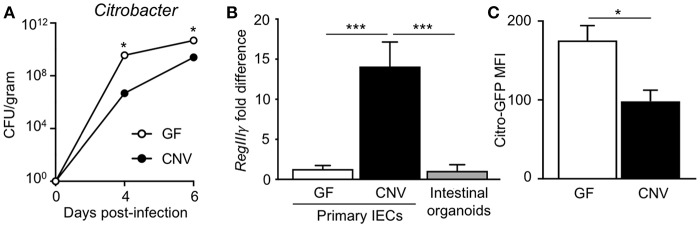
Microbiota exposure decreases pathogen adherence to intestinal epithelial cells. **(A)** *C. rodentium* colony forming units (CFUs) in stool of infected germ-free (GF) and conventionally-housed (CNV) mice during early course of infection (day 4–6). Data are representative of at least 3 independent experiments with 3–4 mice per group. **(B)** Relative RegIIIγ mRNA expression in IECs isolated from the large intestine of GF and CNV mice compared to intestinal organoid cultures. **(C)** MFI of GFP-*C. rodentium* infected intestinal organoids derived from GF and CNV mice. Data represent two independent experiments with 3–4 mice per group. Results are mean ± SEM. ^*^p < 0.05, ^***^p < 0.001.

*Citrobacter rodentium* (*C. rodentium*) is a murine bacterial pathogen with similar pathogenesis to enteropathogenic *E. coli* in humans. Germ-free (GF) mice infected with *C. rodentium* exhibited significantly higher pathogen burdens compared to conventionally-housed (CNV) mice (Figure 1A), indicating that the microbiota enhance defense against pathogenic colonization. Interestingly, microbiota-sensitive protection against infection was detected as early as day 4 post-infection, suggesting the presence of the microbiota affect the initial establishment of *C. rodentium* colonization. Intestinal epithelial cells (IECs) produce antimicrobial peptides and consistent with previous studies (35), the microbiota induced IEC expression of the antimicrobial peptide RegIIIγ that targets bacterial pathogens (Figure 1B) (36). In order to investigate how the microbiota induce epithelial-intrinsic defense, intestinal epithelial organoids that are devoid of immune cells were compared. Interestingly, intestinal epithelial organoids generated from CNV-housed mice expressed significantly reduced levels of RegIIIγ (Figure 1B). However, despite this impairment in RegIIIγ expression, organoids from CNV mice exhibited lower adherent GFP-expressing *C. rodentium* compared to GF organoids as measured by flow cytometry (Figure 1C). These data suggest that other mechanisms, aside from RegIIIγ, contribute to microbiota-sensitive IEC-intrinsic resistance against pathogenic bacterial adherence.

### Microbiota Exposure Downregulates C-Type Lectin 2e Expression in Intestinal Epithelial Cells

**Figure 2:**
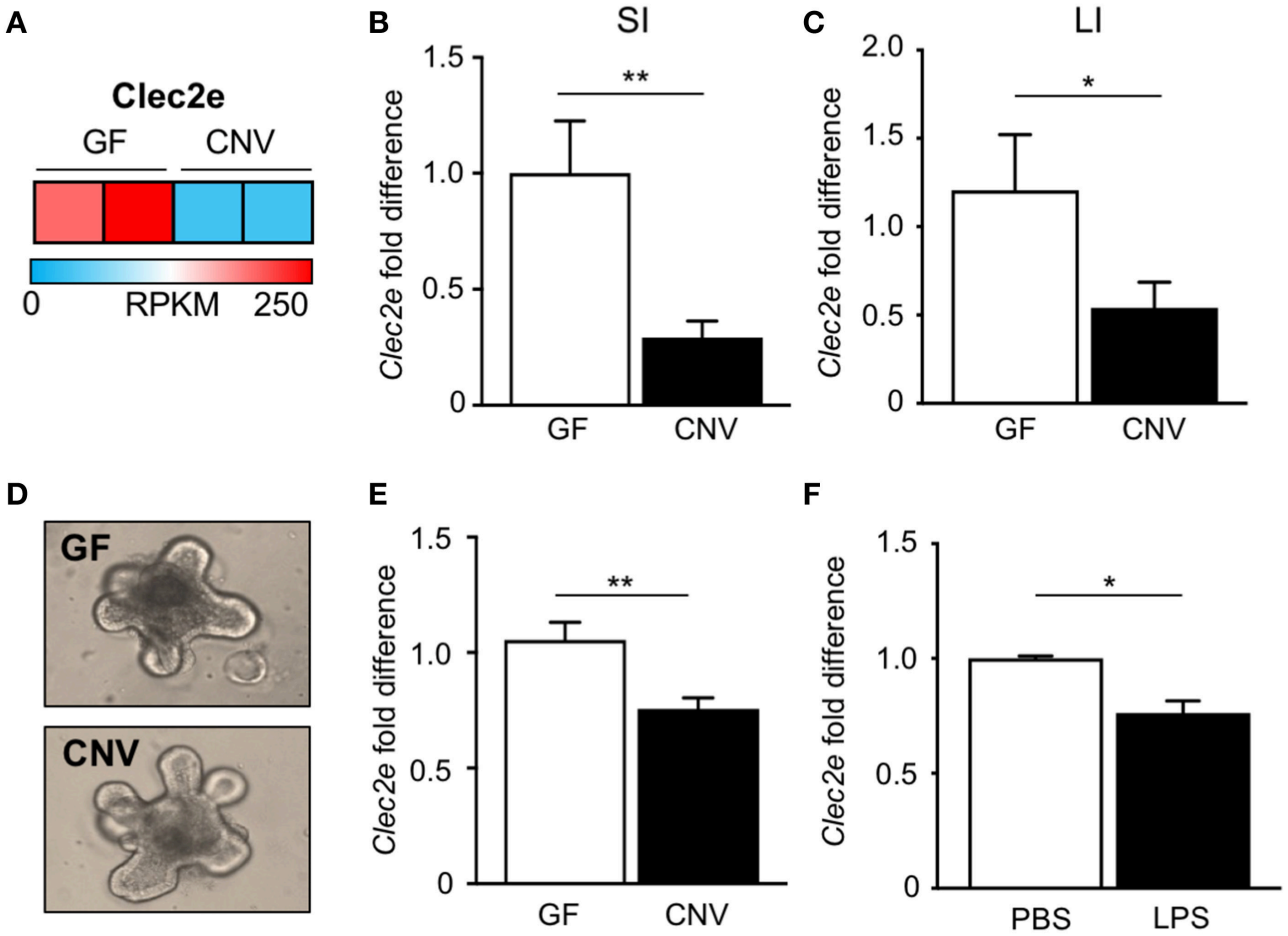
Microbiota downregulate intestinal epithelial expression of C-type lectin Clec2e. **(A)** Relative Clec2e expression levels in IECs harvested from small intestine of GF and CNV mice, represented as reads per kilobase per million mapped reads (RPKM). **(B,C)** Real-time quantitative PCR analysis of Clec2e in IECs from GF and CNV mice isolated from **(B)** small intestine (SI) or **(C)** large intestine (LI). **(D)** Intestinal organoids derived from GF and CNV mice. **(E)** Clec2e mRNA expression in GF and CNV organoids. **(F)** Clec2e mRNA expression in GF intestinal organoids in the absence or presence of LPS. Data represent two independent experiments with 3–4 mice per group. Results are mean ± SEM. ^*^p < 0.05, ^**^p < 0.01.

In order to identify potential mediators of pathogen adherence that are regulated by the microbiota, we compared genes expressed in IECs harvested from the intestine of GF and CNV mice by RNA-sequencing. These analyses identified C-type lectin 2 member e (Clec2e; Clr-a) as one of the most significantly downregulated genes in IECs in response to microbial exposure (Figure 2A). Clec2e expression was confirmed to be decreased by quantitative PCR (qPCR) in IECs from independent cohorts of GF and CNV mice in both the small intestine (Figure 2B) and large intestine (Figure 2C). To investigate whether microbiota-suppressed Clec2e expression was maintained in the absence of immune cells or persistent microbial stimulation, intestinal organoid cultures were generated from colonic crypts isolated from GF and CNV mice (Figure 2D). Consistent with IECs *in vivo*, Clec2e expression was repressed in organoids derived from CNV mice (Figure 2E), suggesting prior exposure to the microbiota led to sustained downregulation of Clec2e. To determine how the microbiota may suppress Clec2e expression, intestinal organoids derived from GF mice were incubated with LPS (Figure 2F). LPS reduced Clec2e expression in IECs, although less than observed in CNV organoids, suggesting that microbial-derived components may collectively regulate epithelial Clec2e expression.

### Expression of Clec2e Increases Cellular Adherence of Enteric Bacterial Pathogens

**Figure 3:**
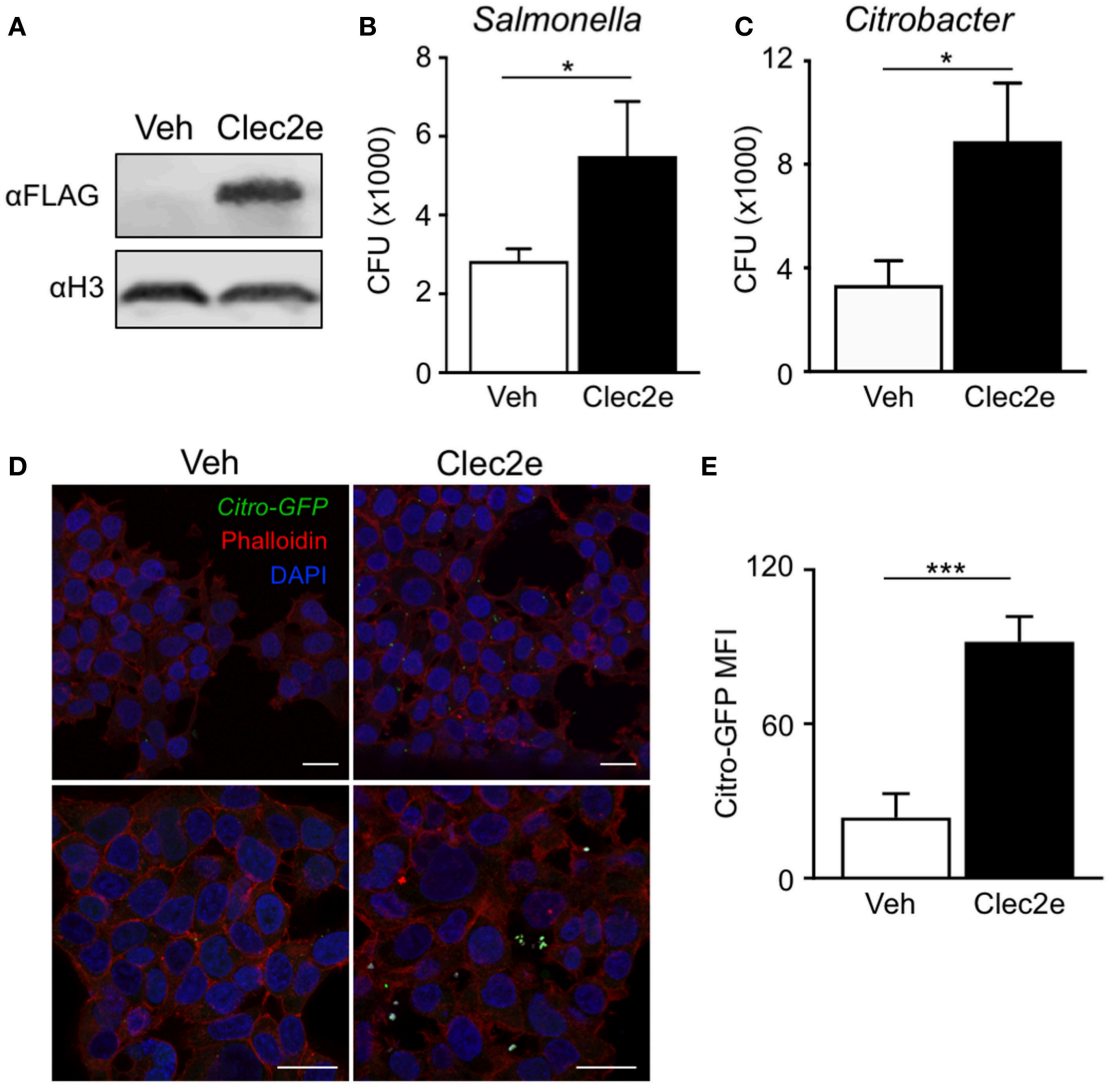
Expression of Clec2e enables increased adherence of bacterial pathogens. Clec2e overexpressed in HEK293T cells using a FLAG-tagged Clec2e expression vector. **(A)** Western blot analysis of Clec2e-FLAG expression in vehicle or Clec2e-FLAG transfected HEK293T cell lysates. **(B,C)** *In vitro* bacterial adhesion assay of infected HEK293T cells, expressed as adherent CFUs 6 h post infection. **(D)** Immunofluorescent staining of HEK293T cells infected with GFP-expressing *C. rodentium* (green). Mammalian cell membrane and nuclei are stained with Phalloidin (red) and DAPI (blue), respectively. Scale bars, 25 μm. **(E)** MFI of GFP-*C. rodentium* bound to vehicle-treated or Clec2e-transfected HEK293T cells. Data represent 2 independent experiments with *n* = 3 per group. Results are mean ± SEM. ^*^p < 0.05, ^***^p < 0.001.

Clec2e is a homodimeric cell surface glycoprotein expressed in the intestinal epithelium that shares homology with other C-type lectins (37, 38). However, unlike other CLEC2 family members, Clec2e does not interact with Nkrp1 receptors (37, 39). C-type lectin receptor family members contain extracellular carbohydrate binding domains that associate with common pathogen-associated molecular patterns including mannose, fucose, and β-glycan (40, 41), provoking the hypothesis that Clec2e may facilitate bacterial adhesion to host cells. To test whether Clec2e plays a role in bacterial adhesion to mammalian cells, Clec2e-FLAG was overexpressed in HEK293T cells followed by incubation with either *Salmonella* or *C. rodentium*, enteric bacterial pathogens that directly bind to IECs. Expression of transfected Clec2e was confirmed by Western analyses (Figure 3A). Interestingly, Clec2e-expressing cells exhibited significantly greater adherence of *Salmonella* (Figure 3B) and *C. rodentium* (Figure 3C) compared to negative control cells. Bacterial adherence of GFP-expressing *C. rodentium* to Clec2e-expressing cells was confirmed at the cellular level by immunofluorescence (Figure 3D) and flow cytometry (Figure 3E). Together, these data indicate that Clec2e expression promotes adherence of bacterial pathogens to mammalian cells.

### Microbiota Induce Loss of Histone Acetylation and HDAC3 Recruitment Within Regulatory Regions of Clec2e

**Figure 4:**
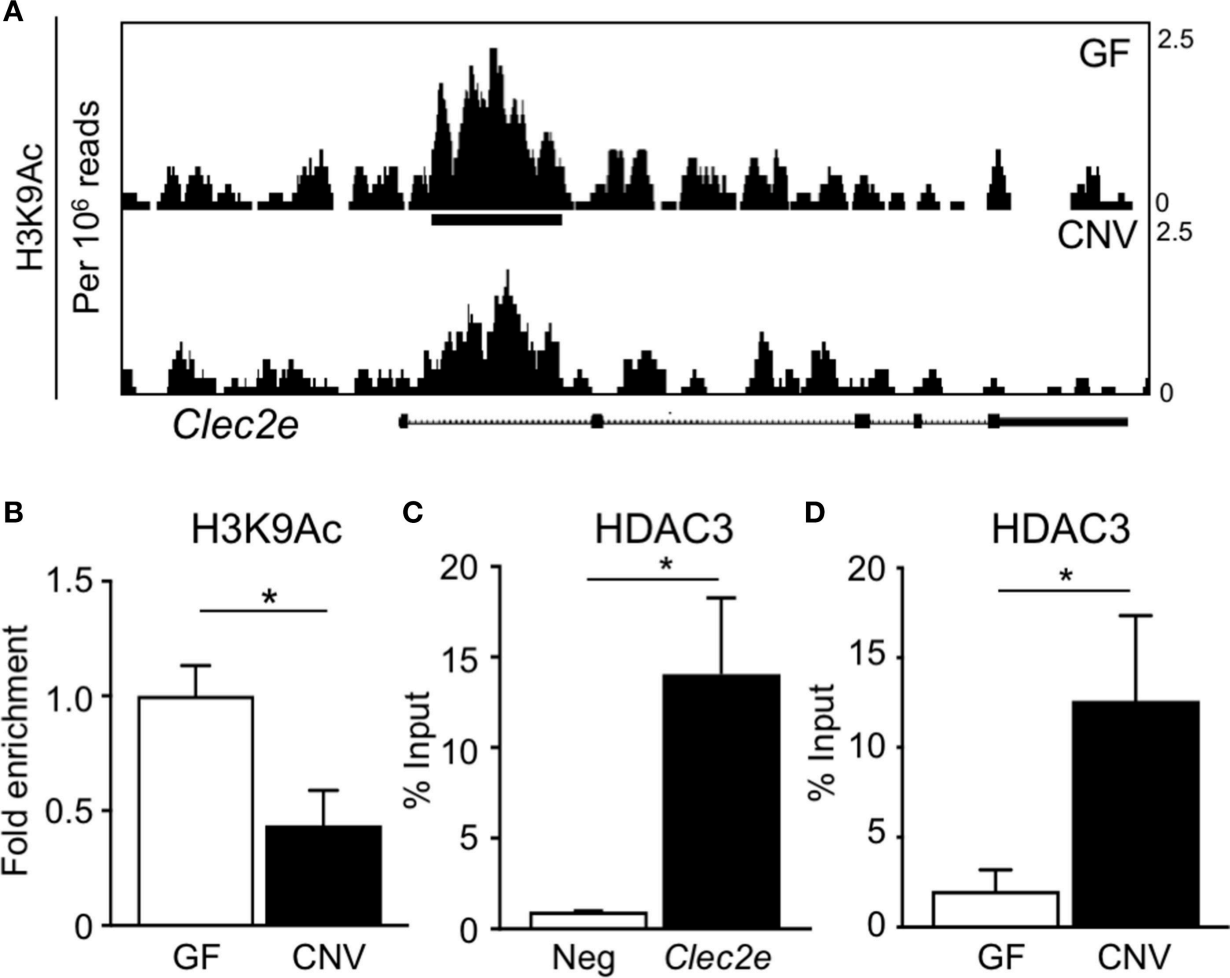
Microbiota induce histone deacetylation and HDAC3 enrichment at Clec2e gene. **(A)** ChIP-seq for H3K9Ac in primary IECs isolated from the small intestine of GF and CNV mice. **(B)** ChIP-qPCR for H3K9Ac in Clec2e from IECs. **(C)** ChIP-qPCR for HDAC3 in Clec2e as percent of input, relative to a negative control gene (Insulin 1). **(D)** HDAC3 ChIP-qPCR in Clec2e from GF and CNV IECs. Data represent two independent experiments with 3-4 mice per group. Results are mean ± SEM. ^*^p < 0.05.

Environmental factors can regulate mammalian gene expression through epigenetic modifications of the chromatin, such as DNA methylation and histone acetylation. Consistent with this, recent studies have revealed that epigenetic pathways may be essential in mediating host-microbe dynamics (17, 42, 43). Therefore, to determine whether the microbiota epigenetically modify chromatin at the Clec2e gene, histone acetylation was compared in primary IECs harvested from GF and CNV mice. For these analyses, chromatin immunoprecipitation (ChIP)-sequencing (seq) was performed for the histone mark H3K9Ac, which is associated with permissive and actively transcribed chromatin (17). Remarkably, ChIP-seq analyses revealed that H3K9Ac levels were significantly decreased at multiple sites within the Clec2e locus in IECs isolated from CNV mice compared to GF controls (Figure 4A). This loss of histone acetylation in regulatory regions of Clec2e due to the microbiota was confirmed by ChIP-qPCR for H3K9Ac (Figure 4B). Previous studies have demonstrated that histone acetylation in IECs can be regulated by epigenetic-modifying enzymes called histone deacetylase (HDACs) (44, 45). The class I histone deacetylase 3 (HDAC3) deacetylates histone H3K9Ac and mediates microbiota-dependent regulation of epithelial gene expression (25, 27). Thus, to determine whether HDAC3 epigenetically regulates Clec2e, HDAC3 recruitment was first examined by ChIP. HDAC3 was enriched at the site of differential H3K9Ac in Clec2e (Figure 4B) relative to a negative non-HDAC3 target (Figure 4C), supporting that Clec2e is a direct target of HDAC3. Interestingly, IECs from CNV mice exhibited significantly higher HDAC3 recruitment to Clec2e compared to IECs from GF mice (Figure 4D). Collectively, these data indicate that the microbiota direct epigenetic regulation of Clec2e through HDAC3.

### HDAC3 Regulates Epithelial Clec2e Expression and Pathogen Adherence

**Figure 5:**
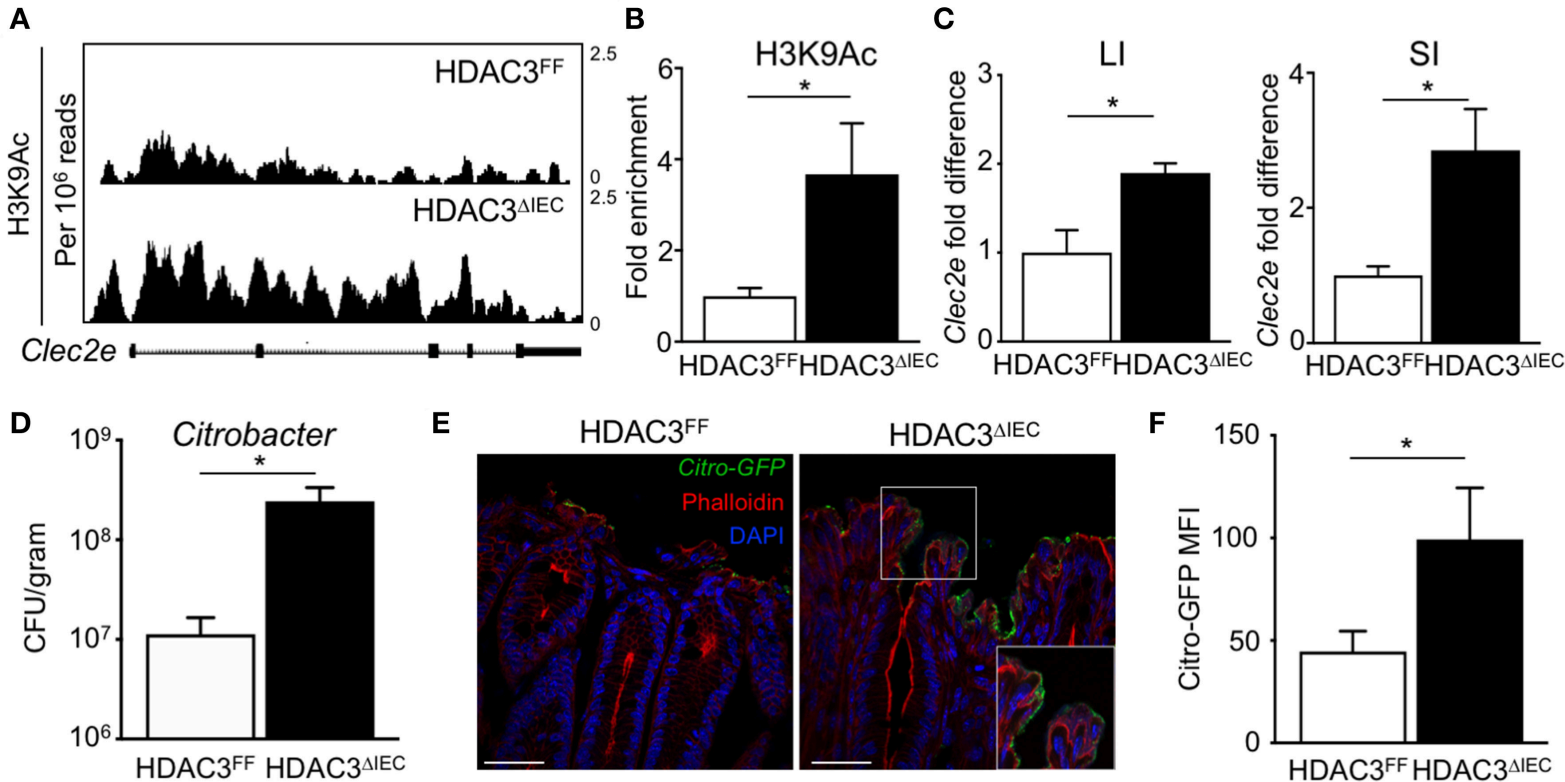
HDAC3 regulates epithelial Clec2e expression and pathogen adherence. **(A)** ChIP-seq for H3K9Ac in primary IECs isolated from the large intestine of HDAC3^FF^ and HDAC3^Δ*IEC*^ mice. **(B)** ChIP-qPCR for H3K9Ac in Clec2e in IECs from the small intestine. **(C)** Clec2e expression in IECs from large and small intestine of HDAC3^FF^ and HDAC3^Δ*IEC*^ mice. **(D)** *C. rodentium* CFUs in stool at day 6 post infection. **(E)** Fluorescence microscopy of colon from HDAC3^FF^ and HDAC3^Δ*IEC*^ mice infected with GFP*-C. rodentium*. (Green: GFP-*C. rodentium*, Red: Phalloidin, Blue: DAPI). Scale bars, 25 μm. **(F)** MFI of GFP*-C. rodentiu*m infected intestinal organoids derived from HDAC3^FF^ and HDAC3^Δ*IEC*^ mice. Data are representative of at least 2 independent experiments with 3–4 mice per group. Results are mean ± SEM. ^*^p < 0.05.

The microbiota-dependent increase in HDAC3 recruitment to Clec2e suggests that loss of H3K9Ac and decreased expression of Clec2e in response to the microbiota could be mediated by HDAC3. Thus, to directly test whether IEC-intrinsic HDAC3 regulates histone acetylation within the Clec2e gene, ChIP-seq for H3K9Ac was performed in IECs harvested from mice that lack HDAC3 expression specifically in IECs (HDAC3^Δ*IEC*^) compared to floxed HDAC3^FF^ control mice (25). Consistent with histone deacetylation by HDAC3, IECs harvested from the large intestine of mice lacking IEC-HDAC3 (HDAC3^Δ*IEC*^) exhibited significantly higher levels of H3K9Ac in Clec2e compared to IECs from HDAC3^FF^ mice (Figure 5A). Increased H3K9Ac enrichment within the microbiota-sensitive regulatory region in the Clec2e gene was also identified in IECs from the small intestine of HDAC3^Δ*IEC*^ mice (Figure 5B), indicating that Clec2e histone acetylation is regulated by epithelial HDAC3. HDAC3 recruitment is associated with transcriptional repression of bound genes. Thus, to test whether elevated H3K9Ac with HDAC3 depletion corresponds with altered expression, Clec2e mRNA expression was measured in IECs from HDAC3^FF^ and HDAC3^Δ*IEC*^ mice. Consistent with the role of HDAC3 as a transcriptional repressor of direct targets, these analyses revealed significantly increased Clec2e expression in IECs harvested from the small and large intestine of HDAC3^Δ*IEC*^ mice compared to IECs from HDAC3^FF^ controls (Figure 5C), indicating that Clec2e expression in HDAC3-deficient IECs results from impaired HDAC3-mediated deacetylation within the Clec2e gene. Collectively, these studies demonstrate that epigenetic and transcriptional regulation of Clec2e broadly depends on epithelial HDAC3 expression in the intestine.

To next test whether HDAC3-dependent regulation of IECs affects pathogen adhesion, HDAC3^FF^ and HDAC3^Δ*IEC*^ mice were infected with *C. rodentium*. Interestingly, *C. rodentium*-infected HDAC3^Δ*IEC*^ mice exhibited higher pathogen burden (Figure 5D) and increased GFP-*C. rodentium* adherence to IECs (Figure 5E) relative to infected HDAC3^FF^ control mice, confirming increased pathogen adhesion in HDAC3^Δ*IEC*^ mice. Further, to test this in the absence of immune cells, intestinal organoids were generated from the colon of control HDAC^FF^ mice and mice lacking HDAC3 in IECs. Consistent with the *in vivo* findings, HDAC3^Δ*IEC*^ organoids exhibited elevated GFP-*C. rodentium* binding compared to cells from floxed controls (Figure 5F). Taken together, these data indicate that regulation of Clec2e expression in IECs by HDAC3-mediated histone deacetylation can promote decreased bacterial pathogen adherence to the intestinal epithelium.

## Discussion

The intestinal epithelium not only maintains intestinal homeostasis to innocuous commensals, but it also defends against invading pathogens (13, 46). Our data indicate that the microbiota can promote epithelial defense by epigenetically suppressing Clec2e-mediated pathogen adherence. Consistent with previously published data (10, 27), we show that GF mice are more susceptible to enteric infection relative to microbiota-replete mice. Previous studies have focused on investigating how the microbiota impact immune cell activation and antibacterial immunity (11, 47, 48), however we observed very early susceptibility to *C. rodentium* infection in GF mice suggesting an important role for innate responses. By employing intestinal organoid cultures, we determined that the microbiota directly impact IEC-intrinsic defense and identified that Clec2e downregulation by the microbiota can reduce pathogen colonization. The microbiota influence several aspects of IEC biology and microbiota-sensitive alterations in IEC composition can also impact host resistance to enteric infection. However, Clec2e is expressed throughout the intestinal epithelium including progenitor and differentiated epithelial cells (37), suggesting that differences in epithelial composition induced by the microbiota are unlikely to be a main mechanism regulating Clec2e expression.

Consistent with our data, studies using GF mice and mouse models that are deficient for pattern recognition receptor mediators have revealed that mucins and antimicrobial peptides require microbial stimulation for expression (35, 49–52). These proteins work in concert to prevent intestinal infection by restricting bacterial adhesion and invasion, limiting microbial growth and colonization, and directly killing bacteria. For example, mucins function by forming a protective barrier that limits access of microbes to underlying IECs and can bind to several enteric pathogens including *C. rodentium* to prevent adhesion (26, 53). In addition, expression of cathelicidin-related antimicrobial peptide by IECs plays an important and non-redundant role in preventing *C. rodentium* adhesion and colonization, especially in early stages of infection (54). Another member of the C-type lectin family with structural similarity to Clec2e, RegIIIγ, binds intestinal bacteria via interactions with peptidoglycan carbohydrates and directly lyses bacteria (35). These studies, combined with our Clec2e data, demonstrate that the microbiota direct multiple IEC-intrinsic host defenses that alter bacterial access to IECs and limit adhesion and colonization.

Similar to enteropathogenic *E. coli* and enterohemorrhagic *E. coli, C. rodentium* employs a type 3 secretion system and other virulence strategies to attach to the apical plasma membrane of IECs (55). *Salmonella* is also equipped with a type 3 secretion system and several fimbriae proteins that enable adherence and invasion to colonic IECs (3, 4, 56). Genetic deletion of type 3 secretion systems or fimbriae molecules drastically reduces bacterial colonization, confirming the necessity of these molecules for pathogenesis (26, 55, 56). Fimbriae and other filopodia-like extensions that enable bacterial adhesion often interact with host plasma membrane associated proteins. *Salmonella* fimbriae bind to a specific glycosylated moiety that is abundantly expressed in the intestinal epithelium (56). Interestingly, Clec2e is a heavily glycosylated protein whose expression is restricted to the intestinal epithelium and is downregulated with LPS or Poly:(IC) stimulation (37), suggesting it may play a functional role in regulating intestinal host defense. Future investigation will require GF and CNV Clec2e^Δ*IEC*^ knockout models in combination with mono-association studies to dissect the contribution of specific commensal microbes or microbial components that influence *in vivo* regulation of pathogen control by Clec2e.

Through global RNA-sequencing analysis we identified a drastic reduction in the expression of the C-type lectin protein, Clec2e, in IECs isolated from CNV mice compared to GF controls. Clec2e (Clr-a) is an orphan C-type lectin molecule that closely resembles the natural killer (NK) gene complex receptor, Clec2h (Clr-f). However, unlike Clec2h, Clec2e does not bind any known NK cell receptors (37, 39, 57). In addition to being signaling partners for NK cell receptors, C-type lectin molecules play a crucial role in recognition of conserved pathogen-associated molecular patterns. Specifically, C-type lectin receptors bind carbohydrate structures commonly associated with microbial cell wall components including mannose, fucose, and β-glucans (35, 40, 41). Further, expression of Clec2e is restricted to the intestinal epithelium and is downregulated with LPS and Poly:(IC) in a TLR3-dependent manner (37). Here, we demonstrate that overexpression of Clec2e is sufficient to promote bacterial adherence to mammalian cells. While the ligand of Clec2e remains unknown, its structural similarities to Dectin-1 and RegIII microbial binding proteins, along with our bacterial adhesion data, suggests Clec2e may bind conserved microbial cell wall components or bacterial pili and fimbria necessary for cellular adherence. Although expanded studies are needed to fully interrogate how Clec2e interacts with commensal bacterial populations and pathogens beyond *Salmonella* and *C. rodentium*, a lack of Clec2e suppression may contribute to heightened susceptibility of GF or antibiotic-treated mice to infection (48).

Epigenetic modifications enable host cells to alter gene expression without modifying the genetic sequence and changes in the host epigenome occur downstream of external environmental signals (20–22). Recent studies, focused predominantly on immune cells types, have supported that the microbiota may imprint or epigenetically prime genes in the host through enzymes such as DNA methyltransferases (58), histone methyltransferases (29, 59), and HDACs (25, 60–63). In addition to HDAC3, other class I HDACs are expressed in IECs (44, 45) and the role of these HDACs as well as other regulatory proteins may further alter epigenetic regulation of the Clec2e gene in IECs. IECs are equipped to sense and respond to common microbial moieties such as LPS, and previous studies showed TLR4 expression in IECs was epigenetically primed by the microbiota (64). Specifically, DNA methylation of TLR4 was decreased in GF mice compared to CNV controls, consistent with reduced TLR4 expression with microbial exposure (64). Histone acetylation is associated with permissive and actively transcribed chromatin. Our data using intestinal organoids revealed that the microbiota mediate durable changes in IECs that remain even after microbial stimulation has been removed. In the presence of the microbiota, H3K9Ac was reduced in the Clec2e gene which directly corresponds with reduced expression of Clec2e in IECs from CNV mice, indicating that the microbiota epigenetically regulate Clec2e expression. This study further demonstrates that H3K9Ac in Clec2e is regulated by HDAC3 as loss of HDAC3 expression leads to increased histone acetylation and loss of transcriptional repression of Clec2e. Taken together, our data demonstrate a novel mechanism by which the microbiota promote host defense through suppression of IEC-intrinsic pathways that are coopted for pathogen adherence and highlights that epigenetic regulation of innate pathways in IECs may represent a potent, long-lasting mechanism by which the microbiota prime host defense.

## Data Availability

The datasets generated for this study can be found in Gene Expression Omnibus (GEO), GSE128362, GSE50453, GSE128369.

## Ethics Statement

All experiments were performed according to the animal guidelines upon approval of the Institutional Animal Care and Use Committee at CCHMC.

## Author Contributions

TA, VW, and EE designed the studies and analyzed the data. VW, EE, TR, and JW carried out experiments. BV provided bacterial strains. TA, VW, and EE wrote the manuscript.

### Conflict of Interest Statement

The authors declare that the research was conducted in the absence of any commercial or financial relationships that could be construed as a potential conflict of interest.
